# Hyperoxia Induces Ferroptosis and Impairs Lung Development in Neonatal Mice

**DOI:** 10.3390/antiox11040641

**Published:** 2022-03-26

**Authors:** Hsiu-Chu Chou, Chung-Ming Chen

**Affiliations:** 1 Department of Anatomy and Cell Biology, School of Medicine, College of Medicine, Taipei Medical University, Taipei 110, Taiwan; chou0217@tmu.edu.tw; 2 Department of Pediatrics, Taipei Medical University Hospital, Taipei 110, Taiwan; 3 Department of Pediatrics, School of Medicine, College of Medicine, Taipei Medical University, Taipei 110, Taiwan

**Keywords:** hyperoxia, glutathione, glutathione peroxidase 4, malondialdehyde, mean linear intercept, von Willebrand factor

## Abstract

Oxygen is often required to treat newborns with respiratory disorders, and prolonged exposure to high oxygen concentrations impairs lung development. Ferroptosis plays a vital role in the development of many diseases and has become the focus of treatment and prognosis improvement for related diseases, such as neurological diseases, infections, cancers, and ischemia-reperfusion injury. Whether ferroptosis participates in the pathogenesis of hyperoxia-induced lung injury remains unknown. The aims of this study are to determine the effects of hyperoxia on lung ferroptosis and development in neonatal mice. Newborn C57BL/6 mice were reared in either room air (RA) or hyperoxia (85% O_2_) at postnatal days 1–7. On postnatal days 3 and 7, the lungs were harvested for histological and biochemical analysis. The mice reared in hyperoxia exhibited significantly higher Fe^2+^, malondialdehyde, and iron deposition and significantly lower glutathione, glutathione peroxidase 4, and vascular density than did those reared in RA on postnatal days 3 and 7. The mice reared in hyperoxia exhibited a comparable mean linear intercept on postnatal day 3 and a significantly higher mean linear intercept than the mice reared in RA on postnatal day 7. These findings demonstrate that ferroptosis was induced at a time point preceding impaired lung development, adding credence to the hypothesis that ferroptosis is involved in the pathogenesis of hyperoxia-induced lung injury and suggest that ferroptosis inhibitors might attenuate hyperoxia-induced lung injury.

## 1. Introduction

Oxygen is often required to treat newborns with respiratory disorders. However, prolonged exposure to high concentrations of oxygen leads to lung inflammation and acute lung injury [1,2]. Neonatal rodents with prolonged hyperoxia exhibited increased lung inflammation and impaired alveolarization and angiogenesis that are comparable to the pathologies of human bronchopulmonary dysplasia (BPD) [3,4]. The pathogenesis of BPD is multifactorial and characterized by the arrest of alveolar and vascular growth associated with inflammation [5,6]. The global incidence of BPD was reported to be 17–75% in infants born before 28 weeks of gestation [7]. BPD is an essential cause of morbidity and mortality in preterm infants, even with ideal ventilation policies, an increased use of noninvasive ventilation, and the early administration of surfactant [8]. Many infants with BPD are left with significant respiratory morbidity, including reactive airway dysfunction and the development of obstructive lung disease during childhood [9,10]. The lungs of term newborn mice are structurally similar to the lungs of human infants born at 26–28 weeks of gestation [11]. Therefore, newborn mice offer a useful model for the study of lung injury and lung development.

Prolonged hyperoxic exposure causes the generation of reactive oxygen species, increases apoptotic signaling, and results in hyperoxia-induced lung injury [12,13]. Unlike apoptosis, ferroptosis is an iron-dependent, nonapoptotic cell death modality characterized by the accumulation of lipid hydroperoxides and reactive oxygen species in cellular membranes [14,15]. Ferroptosis is determined using the balance between iron accumulation-induced lipid reactive oxygen species production and the antioxidant system that prevents lipid peroxidation [16]. Glutathione peroxidase 4 (GPX4) is the major protective mechanism against peroxidation damage, is the central regulator of ferroptosis, and is often regarded as the maker of ferroptosis [17]. Glutathione (GSH) deprivation can result in ferroptosis and GPX4 inactivation. The distinctive features of ferroptosis are mitochondria shrinkage with increased membrane density and reduced mitochondrial cristae, which are different from other modes of cell death. Recent studies have revealed that ferroptosis plays a vital role in the development of many diseases; ferroptosis has become the focus of research on the treatment and prognosis improvement of related diseases, such as neurological diseases, infections, cancers, and ischemia-reperfusion injury [18]. Jia et al. reported that ferroptosis may be involved in the pathological process of hyperoxic lung injury in neonatal rats exposed to hyperoxia (85%) for 7 days [19]. However, the successive effects of hyperoxia on ferroptosis components have not been investigated. Thus, in this study, we used a hyperoxia-induced lung injury model to determine the successive effects of hyperoxia exposure on lung ferroptosis and alveolar and vascular development on postnatal days 3 and 7 in neonatal mice.

## 2. Materials and Methods

### 2.1. Experimental Groups

We conducted the experiments in accordance with the guidelines and regulations of the Institutional Animal Care and Use Committee of Taipei Medical University. Time-dated pregnant C57BL/6 mice were housed in individual cages with free access to laboratory food and water. A 12:12-h light–dark cycle was maintained. The mice were allowed to deliver vaginally at term. Within 12 h of birth, the litters were pooled and randomly redistributed among the newly delivered mothers, and the pups were then randomly assigned to be reared in room air (RA) or O_2_-enriched air. The pups in the hyperoxia (O_2_, normobaric) group were reared in an atmosphere containing 85% O_2_ during postnatal days 1–7. The pups in the RA group were reared in RA during postnatal days 1–7. To prevent O_2_ toxicity in the nursing mothers, we rotated them between the O_2_ treatment and the RA control litters every 24 h. An O_2_-rich (85%) atmosphere was maintained in a transparent 40 × 50 × 60 cm^3^ plexiglass chamber, which received a continuous inflow of O_2_ at 4 L/min. The levels of O_2_ were monitored using a ProOx P110 monitor (NexBiOxy, Hsinchu, Taiwan). On postnatal days 3 and 7, the mice pups were anesthetized with 1% isoflurane (Halocarbon Laboratories, River Edge, NJ, USA) and their lungs were harvested for histological and biochemical analysis.

### 2.2. Measurement of Ferroptosis Indicators

Ferroptosis is caused by iron accumulation and lipid peroxidation [20]. Malondialdehyde (MDA) is a final product of lipid peroxidation [21]. GPX4, a phospholipid hydroperoxidase, can suppress lipid peroxidation [17]. GSH is an antioxidant compound, and its depletion activates lipoxygenases and inhibits GPX4 activity to induce lipid peroxidation [22]. Lung tissues were homogenized, sonicated, and centrifuged at 500× *g* for 20 min at 4 °C to remove cellular debris. Then the biomarkers of ferroptosis Fe^2+^, MDA, GSH level, and GPX4 activity in lung tissues were assessed using an iron assay kit (catalog number E-BC-K139-M, Elabscience, Houston, TX, USA), MDA assay kit (catalog number MBS2605193, MyBioSource, San Diego, CA, USA), GSH assay kit (catalog number MBS267424, MyBioSource), and GPX4 enzyme-linked immunosorbent assay kit (catalog number MBS934198, MyBioSource), respectively, according to their respective manufacturer’s instructions.

### 2.3. Tissue Preparation for Transmission Electron Microscope

The ultrastructure of the mitochondria of the lung tissue was analyzed with transmission electron microscopy. The lungs were immersed and fixed in 2% paraformaldehyde and 2.5% glutaraldehyde in 0.1 M cacodylate buffer for 1 day. The lung tissues were then minced into 1 mm3 blocks and post-fixed in 1% osmium tetroxide for 1 h. Dehydration in a concentration gradient of ethanol alcohols were followed. Finally, the blocks were embedded in Epon resin and then stained with lead citrate and uranyl acetate. Ultrathin sections (50–60 nm) were double-stained with uranyl acetate and lead citrate and then examined using a Hitachi HT7700 transmission electron microscope (Tokyo, Japan).

### 2.4. Detection of Iron Deposition

An iron staining kit based on the Prussian blue reaction (ScyTek Laboratories, Logan, UT, USA) was used to detect iron deposition in lung tissues. After deparaffinization and rehydration, the 5-µm lung sections were treated with equal volumes of 2% hydrochloric acid solution and potassium ferrocyanide solution for 5 min. The sections were then counterstained with nuclear fast red solution for 5 min, dehydrated with alcohol, cleared with xylene, and mounted with coverslips for further observation. This staining is such that ferric iron shows up as blue and nuclei as red. Positive cells were scored in 4 fields randomly selected from each section using a light microscope and the results were expressed as positive staining cells per high-power field (magnification 400×).

### 2.5. Lung Histology

The lung tissue was immersed with 4% paraformaldehyde in 0.1 M phosphate buffer (pH 7.4) at 4 °C for 24 h. The tissues were then dehydrated in alcohol, cleared in xylene, and embedded in paraffin. Furthermore, 5-µm sections were stained with hematoxylin and eosin, examined using light microscopy, and assessed for lung morphometry. The mean linear intercept (MLI), an indicator of mean alveolar diameter, was assessed in 10 nonoverlapping fields [23]. Following the recommendations of the American Thoracic Society Official workshop report [24,25], we examined inflammatory cells in lung tissue, including neutrophils, small lymphocytes, and alveolar macrophages, suggesting the presence of inflammatory response in hyperoxia-induced lung injury.

### 2.6. Immunohistochemistry of von Willebrand Factor and Vascular Endothelial Growth Factor

Immunohistochemical staining was performed on 5-μm paraffin sections using immunoperoxidase visualization. After routine deparaffinization, heat-induced epitope retrieval was performed by immersing the slides in a 0.01 M sodium citrate buffer (pH 6.0). To block endogenous peroxidase activity and nonspecific binding of antibodies, the sections were preincubated for 1 h at room temperature in 0.1 M PBS containing 10% normal goat serum and 0.3% H_2_O_2_. The sections were then incubated for 20 h at 4 °C with rabbit polyclonal antivWF antibodies (1:100; Abcam, Cambridge, MA, USA) and rabbit polyclonal antivascular endothelial growth factor (VEGF) antibodies (1:50; Santa Cruz Biotechnology, Santa Cruz, CA, USA) as primary antibodies. The sections were then treated for 1 h at 37 °C with biotinylated goat antimouse or rabbit IgG (1:200, Jackson ImmunoResesarch Laboratories Inc., West Grove, PA, USA). Following the reaction produced using reagents from an avidin–biotin complex kit (Vector Laboratories, Burlingame, CA, USA), the reaction products were visualized using a diaminobenzidine substrate kit (Vector Laboratories, Inc.) according to the manufacturer’s recommendations. Microvessel density was determined by counting the vessels with the positive vWF stained in an unbiased manner and by examining a minimum of four random lung fields at ×400 magnifications [26].

### 2.7. Western Blotting of VEGF and GPX4

Lung tissues were homogenized in ice-cold buffer containing 50 mmol/L Tris·HCl (pH 7.5), 1 mmol/L ethylene glycol tetraacetic acid, 1 mmol/L ethylenediaminetetraacetic acid, and a protease inhibitor cocktail (complete mini-tablets; Roche, Mannheim, Germany). Proteins (30 µg) were resolved on 12% sodium dodecyl sulfate–polyacrylamide gel electrophoresis under reducing conditions and electroblotted to a polyvinylidene difluoride membrane (Immobilon P, Millipore). After being blocked with 5% non-fat dry milk, the membranes were incubated with anti-GPX4 (1:750; SC-7269, Santa Cruz Biotechnology), anti-VEGF (1:750; SC-7269, Santa Cruz Biotechnology), and anti-β-actin (1:1000; C4 sc-47778, Santa Cruz Biotechnology) and then incubated with horseradish peroxidase-conjugated goat antimouse (Pierce Biotechnology, Rockford, IL, USA). Protein bands were detected using the BioSpectrum AC System from Pierce.

### 2.8. Lung Cytokine Assay

Approximately 100 mg of lung tissue from each pup was homogenized, sonicated, and centrifuged at 500× *g* for 20 min at 4 °C to remove cellular debris according to the manufacturer’s instructions. The levels of interleukin-6 (IL-6) in the supernatants were determined using the enzyme-linked immunosorbent assay kit (R&D systems, Abingdon, UK).

### 2.9. Statistical Analysis

Data are expressed in terms of the mean ± SD. This study was completed using data from two separate experiments. Age groups were compared using Student’s *t* test. Differences were considered significant at *p* < 0.05.

## 3. Results

The body weights were comparable between the RA and hyperoxia groups on postnatal day 3. On postnatal day 7, mice reared in a hyperoxic environment exhibited a significantly lower body weight than mice reared in RA (Table 1).

### 3.1. Hyperoxia Induced Ferroptosis

**Figure 1:**
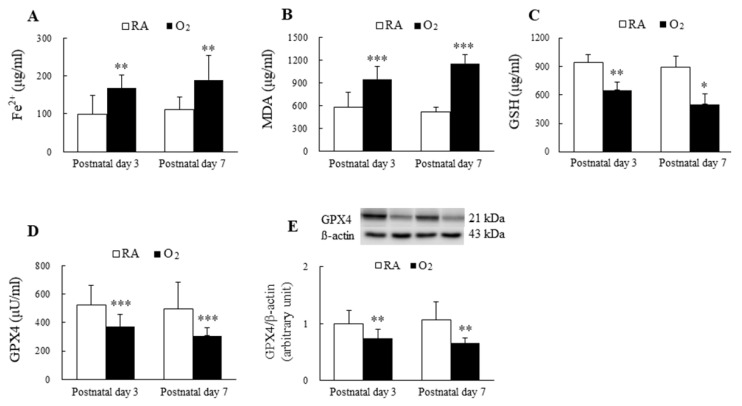
Hyperoxia induced ferroptosis in mice lungs. The ferroptosis level is evaluated through the detection of the biomarkers of ferroptosis. (**A**) Fe^2+^ level, (**B**) MDA level, (**C**) GSH level, (**D**) GPX4 activity, and (**E**) GPX4 protein expression. The mice reared in hyperoxia (*n* = 7–10) exhibited significantly higher Fe^2+^ and MDA levels and significantly lower GSH and GPX4 compared with the mice exposed to RA (*n* = 11). * *p* < 0.05, ** *p* < 0.01, *** *p* < 0.001.

The mice reared in hyperoxia from birth to postnatal days 3 and 7 exhibited significantly higher Fe^2+^ and MDA levels and significantly lower GSH and GPX4 levels compared with the mice exposed to RA on postnatal days 3 and 7 (Figure 1A–E).

### 3.2. Hyperoxia Induced Mitochondrial Morphology Changes

**Figure 2:**
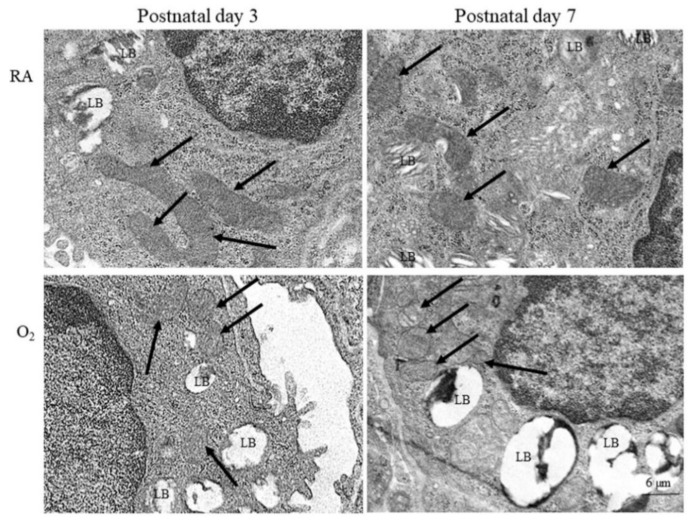
Representative photomicrographs of transmission electron microscopy in mice lung tissue. The alveolar type II epithelial cells of hyperoxia group exhibited abnormal mitochondrial morphology (black arrow) typical of ferroptosis, including shrunken mitochondria, increase density and rupture of membranous structure, and decrease mitochondrial cristae. LB, lamella body.

Representative electron microscope pictures of mice lung tissue are presented in Figure 2. The alveolar type II epithelial cells of hyperoxia group exhibited abnormal mitochondrial morphology typical of ferroptosis, including shrunken mitochondria, increase density and rupture of membranous structure, and decrease mitochondrial cristae on postnatal days 3 and 7. The mice reared in RA displayed no abnormal mitochondrial morphology on postnatal days 3 and 7.

### 3.3. Hyperoxia Increased Iron Deposition

**Figure 3:**
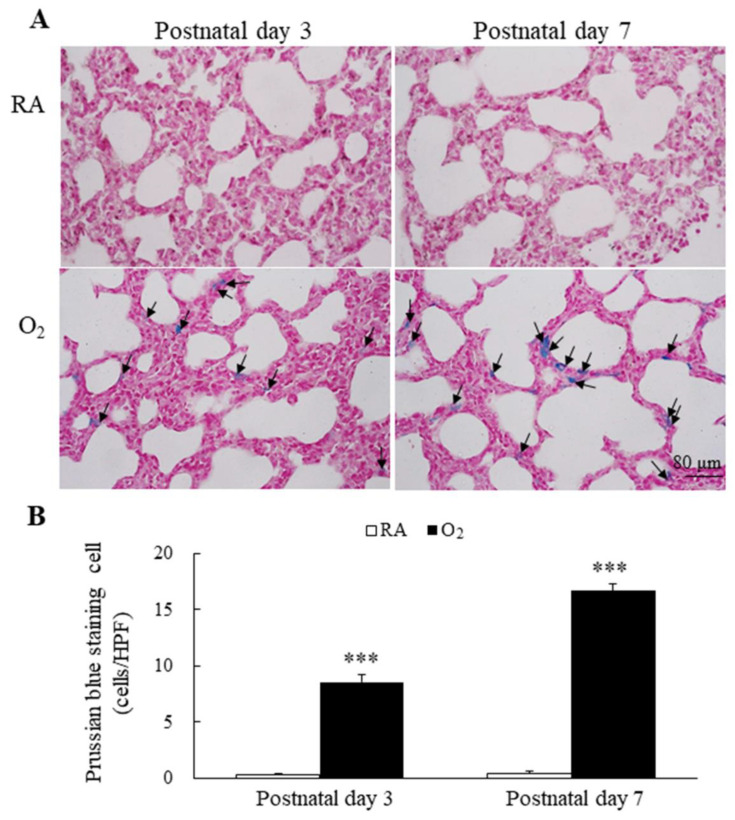
(**A**) Representative photomicrographs of Prussian blue staining and (**B**) the positive cells per high-power field in lung tissue. Prussian blue staining was mainly localized in type II alveolar cells and alveolar macrophages (black arrow). The mice reared in hyperoxia exhibited a significantly higher Prussian blue positive staining cells per high-power field than the mice reared in RA. *n* = 6 mice at each postnatal day. *** *p* < 0.001.

Representative lung sections stained with Prussian blue are presented in Figure 3A. Prussian blue staining was mainly localized in cytoplasm of type II alveolar cells and alveolar macrophages. The mice reared in RA displayed no detectable iron on postnatal days 3 and 7. The mice reared in hyperoxia exhibited a significantly higher Prussian blue positive staining cells per high-power field than the mice reared in RA on postnatal days 3 and 7 (Figure 3B). 

### 3.4. Hyperoxia Impaired Alveolarization

**Figure 4:**
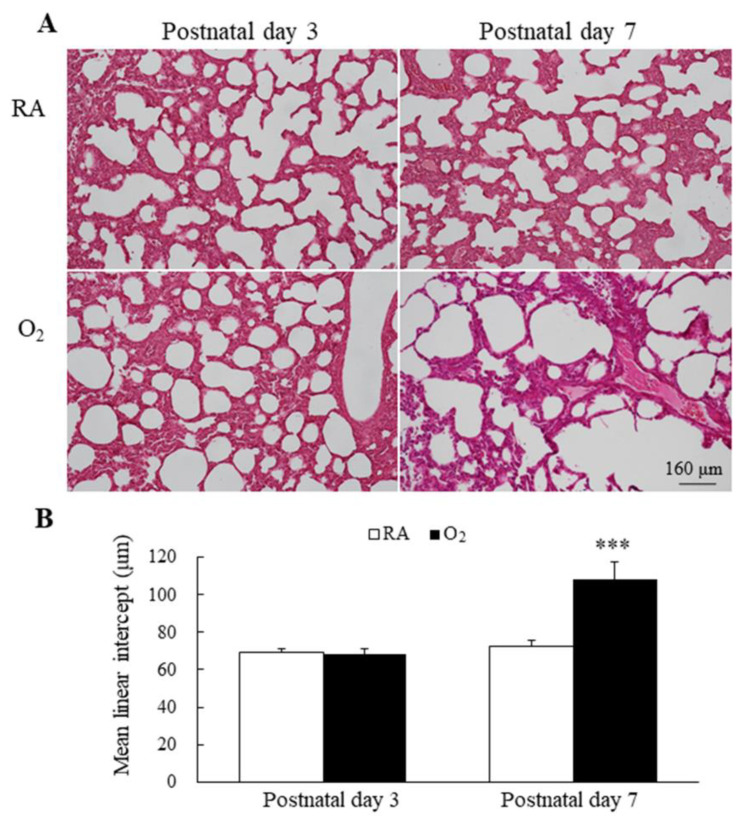
(**A**) Representative H&E-stained lung sections and (**B**) MLI in mice lungs. The mice reared in RA and hyperoxia exhibited comparable MLI on postnatal day 3 and the mice reared in hyperoxia from birth to postnatal day 7 exhibited a significantly higher MLI compared with the mice reared in RA. *n* = 7 mice at each postnatal day. *** *p* < 0.001.

Representative lung sections stained with hematoxylin and eosin are presented in Figure 4A. MLI is an indicator of alveolarization. The mice reared in RA and hyperoxia exhibited comparable MLI on postnatal day 3, and the mice reared in hyperoxia from birth to postnatal day 7 exhibited a significantly higher MLI compared with the mice reared in RA (Figure 4B). 

### 3.5. Hyperoxia Reduced Angiogenesis

**Figure 5:**
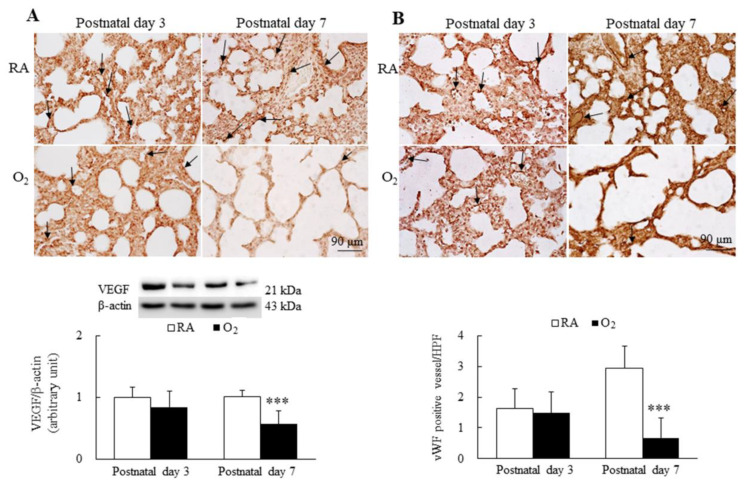
Representative immunohistochemistry and representative Western blots for (**A**) VEGF and (**B**) representative immunohistochemistry of vWF and semiquantitative analysis for vascular density in mice. The mice reared in RA and hyperoxia exhibited similar VEGF (black arrow) and vWF (black arrow) immunoreactivity on postnatal day 3, and the mice reared in hyperoxia exhibited a significantly lower VEGF and protein expression and a significantly lower vWF immunoreactivity and vascular density (black arrow) than the mice reared in hyperoxia on postnatal day 7. *n* = 7 mice at each postnatal day. *** *p* < 0.001.

The representative immunohistochemistry for VEGF and vWF is presented in Figure 5A,B, respectively. VEGF immunoreactivities were primarily detected in the endothelial and epithelial cells. The mice reared in RA and hyperoxia exhibited similar VEGF immunoreactivity and protein expression on postnatal day 3. The mice reared in hyperoxia from birth to postnatal day 7 exhibited significantly lower VEGF immunoreactivity and protein expression than the mice reared in RA. The vWF immunoreactivities were primarily detected in the endothelial cells. The mice reared in RA and hyperoxia exhibited similar vWF immunoreactivity and vascular density on postnatal day 3. The mice reared in hyperoxia from birth to postnatal day 7 exhibited significantly lower vWF immunoreactivity and vascular density than the mice reared in hyperoxia. 

### 3.6. Hyperoxia Induced Lung Inflammation and Increased Cytokine

**Figure 6:**
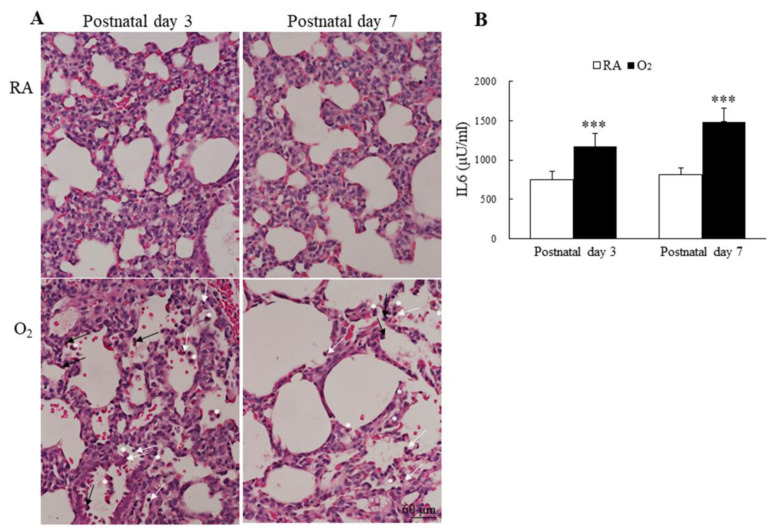
(**A**) Representative H&E-stained lung sections and (**B**) lung IL-6 levels. The mice reared in hyperoxia exhibited numerous inflammatory cells in the alveolar space, neutrophils (black arrow), small lymphocyte (white arrow), and alveolar macrophage (white asterisks). The mice reared in hyperoxia exhibited a significantly higher lung IL-6 levels than the mice reared in RA. *n* = 7–11 mice at each postnatal day. *** *p* < 0.001.

The inflammatory cells, neutrophils, small lymphocytes, and monocytes derived alveolar macrophages were observed more numerous in alveolar space of mice reared in hyperoxia on postnatal days 3 and 7 (Figure 6A). The mice reared in hyperoxia exhibited a significantly higher lung IL-6 levels than the mice reared in RA on postnatal days 3 and 7 (Figure 6B).

## 4. Discussion

Apoptosis activates caspase proteases and is an energy-dependent process of cell death [27]. Necroptosis is a caspase-independent form of cell death [28]. Autophagy is the natural, conserved degradation that removes unnecessary or dysfunctional components of the cell [29]. Ferroptosis is an iron dependent, non-apoptotic mode of cell death, characterized by different cell morphology and function with necrosis, apoptosis, and autophagy [18]. Preclinical studies have demonstrated that hyperoxia activates apoptosis, necroptosis, and autophagy in lung tissues [30,31,32]. However, the consecutive effects of hyperoxia on ferroptosis components have not been investigated.

Our in vivo model demonstrated that hyperoxia exposure during the first 7 days after birth induced ferroptosis on postnatal days 3 and 7 and impaired lung development on postnatal day 7, as evidenced by the increased Fe^2+^ and MDA and decreased GSH, GPX4, and vascular density. The MLI in the RA and hyperoxia groups were comparable on postnatal day 3 and was significantly higher in the hyperoxia group than in the RA group on postnatal day 7. These findings demonstrated that ferroptosis is closely related to hyperoxia-induced lung injury and suggest that ferroptosis is induced at a time point preceding impaired lung development, contributes to the pathogenesis of hyperoxia-induced lung injury, and can be a novel therapeutic target of hyperoxia-induced lung damage. 

In this study, we measured the level of MDA, which is the main end product of lipid peroxidation, because ferroptosis is activated by lipid peroxidation in cells. The MDA level of the hyperoxia-exposed group was higher than that of the RA group on postnatal days 3 and 7. In general, the accumulation of lipid reactive oxygen species is caused by the reduction of the intracellular antioxidant GSH. Thus, we measured the GSH level. We detected decreased levels of GSH in mice reared in hyperoxia on postnatal days 3 and 7. Because a previous study reported that GSH depletion leads to GPX inactivation [33], we measured GPX4 expression. According to the results from the enzyme-linked immunosorbent assay kit and Western blotting procedure, GPX4 protein expression was reduced in the mice reared in hyperoxia on postnatal days 3 and 7. These results indicated that hyperoxia exposure decreased GSH, reduced GPX4 expression, and induced ferroptosis in neonatal mice.

In this study, we observed that hyperoxia exposure from birth to postnatal day 3 induced ferroptosis but not impaired lung development and that hyperoxia from birth to postnatal day 7 induced ferroptosis and impaired lung development in neonatal mice. These findings indicate that ferroptosis was initially induced prior to impaired lung development. These results further demonstrate that ferroptosis contributes to the pathogenesis of hyperoxia-induced lung injury.

The distinguishing features among ferroptosis, apoptosis, autophagy, and necroptosis are small mitochondria with increased mitochondrial membrane densities, a reduction of mitochondria crista, a rupture in the outer mitochondrial membrane, and the presence of a normal nucleus. Both short exposure and long exposure to hyperoxia induced mitochondrial dysregulation and dysfunction and arrested alveolar development in neonatal mice [34,35]. However, whether ferroptosis contributes to the pathogenesis of hyperoxia-induced lung injury remains unknown. In this study, we observed that long exposure to hyperoxia altered mitochondrial morphology, induced ferroptosis, and impaired alveolar development. These results further indicate the crucial role of ferroptosis in hyperoxia-induced lung injury.

## 5. Conclusions

This study demonstrated that hyperoxia exposure during the first 7 days after birth induced ferroptosis, as indicated by increased Fe^2+^ and MDA and decreased GSH and GPX4. Ferroptosis was induced at a time point preceding impaired lung development, adding credence to that ferroptosis is involved in the pathogenesis of hyperoxia-induced lung injury and suggest that ferroptosis inhibitors might attenuate hyperoxia-induced lung injury. Currently, no effective therapy for hyperoxia-induced lung injury is available. These results suggest that ferroptosis may be a potential therapeutic target against hyperoxia-induced lung injury.

## Figures and Tables

**Table 1:** Body weight on postnatal days 3 and 7 in the room air- or hyperoxia-reared mice.

Treatment	*n*	Body Weight on Postnatal Day 3 (g)	*n*	Body Weight on Postnatal Day 7 (g)
Room air	11	2.23 ± 0.35	11	3.28 ± 0.56
Hyperoxia	10	2.13 ± 0.29	7	2.64 ± 0.41 *

Values are presented as mean ± standard deviation. * *p* < 0.05, compared with the room air group.

## Data Availability

Data is contained within the article.
